# Acute Agitation as an Initial Manifestation of Neuro-Behçet's Disease

**DOI:** 10.1155/2018/5437027

**Published:** 2018-11-29

**Authors:** Yuki Otsuka, Tetsuya Yumoto, Hiromi Ihoriya, Namiko Matsumoto, Kota Sato, Koji Abe, Hiromichi Naito, Atsunori Nakao

**Affiliations:** ^1^Department of Emergency and Critical Care and Disaster Medicine, Okayama University Graduate School of Medicine, Dentistry and Pharmaceutical Sciences, Japan; ^2^Center for Graduate Medical Education, Okayama University Hospital, Japan; ^3^Department of Neurology, Graduate School of Medicine, Dentistry and Pharmacy, Okayama University, Japan

## Abstract

Managing acutely agitated or violent patients in the emergency department (ED) represents a significant challenge. Acute agitation as an initial manifestation of neuro-Behcet's disease (NBD) is an extremely rare clinical entity. A 44-year-old male, who had been complaining about a severe headache and fever for several days, was admitted to our ED due to acutely presented incontinence and agitation. On admission, physical restraint and sedation with sevoflurane and propofol were required for his combative and violent behavior. Cerebrospinal fluid examination revealed increased cell count. Fluid attenuated inversion recovery magnetic resonance imaging showed a high intensity signal in the left parietal lobe and bilateral occipital lobe. As infectious meningoencephalitis was suspected, empirical therapy was immediately started. He recovered uneventfully without neurological defect in seven days. Based on positive human leukocyte antigen B-51 and clinical manifestations, the diagnosis of NBD was made and remitted by steroid therapy. Although acute NBD commonly presents with focal neurological symptoms, psychiatric symptoms could be considered the first manifestation. A focused and thorough examination coupled with appropriate management strategies can assist emergency clinicians safely and effectively manage these patients.

## 1. Introduction

Acute agitation, a common but difficult condition encountered in the emergency department (ED), has a very broad range of differential diagnoses that include neurologic, metabolic, toxicologic, psychiatric, and infectious causes [1]. Therefore, emergency physicians need to rapidly and appropriately assess and manage patients with this condition, as the missing of signs of acute agitation may result in a poor prognosis.

The present case report aims to describe the signs, symptoms, differential diagnosis, treatment, and course of a 44-year-old male patient with neuro-Behçet's disease (NBD) presenting acute agitation as an initial manifestation. Behçet's disease is a chronic, vasculitic, multisystem syndrome consisting of recurrent painful oral and genital ulcers and relapsing uveitis. About 5% of adult patients experience neurologic symptoms at the onset of Behçet's disease [2, 3].

To the best of our knowledge, acute psychotic attack is extremely rare as an initial symptom of NBD and only a few reports have documented acute psychiatric episodes associated with NBD [4, 5]. Although NBD frequently presents with focal neurological signs, psychiatric manifestations could be considered the first symptoms of the disease. With this report, we hope to raise awareness among emergency physicians of the importance of assessing agitation and that agitation may occur as the first manifestation of autoimmune disorders, including NBD.

## 2. Case Presentation

A 44-year-old previously healthy male was taken to our ED by ambulance with acute agitation and fever. He had no family or personal history of psychiatric issues or psychosocial stressors that could have caused a psychotic episode. His family explained that he had been complaining for three days about a severe headache and fever and had acutely presented incontinence, agitation, and violent behavior two hours prior to the visit.

**Figure 1:**
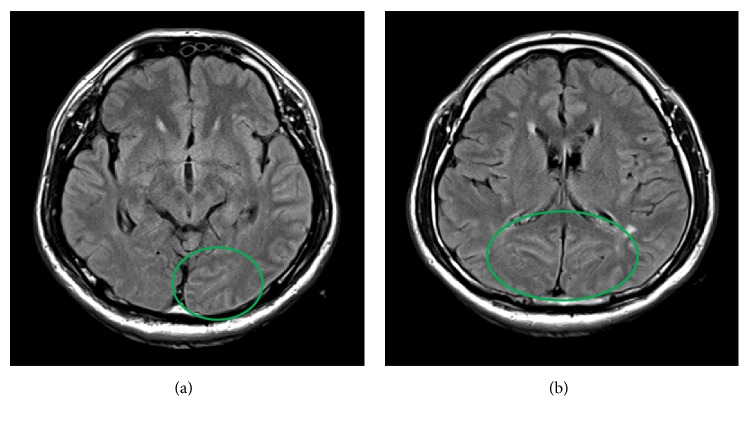
Circled region showing increased T2 signal of sulci on fluid attenuated inversion recovery sequence involving the left parietal lobe (a) and bilateral occipital lobe (b).

On admission, the patient displayed bizarre, incorporative and violent behavior against medical staff. Since nonphysical strategies against agitated behavior referred to as “verbal deescalation” were unsuccessful, we required immediate action to protect the patient as well as medical staff from imminent harm using deep sedation. The Richmond Agitation Sedation Scale was graded as +4. Physical restraint and administration of sevoflurane and propofol allowed for further assessment assuring the safety of the patient and medical staff. His vital signs were remarkable for an isolated fever of 37.8°C associated with Kernig's sign. The patient did not have oral or genital ulcerations or an erythematous rash. His blood test results showed elevated white blood cell count of 16,990/*μ*L with 80.1% neutrophils and C-reactive protein of 19.05 mg/dl. Blood gas analysis showed metabolic acidosis with a pH of 7.306, base excess -6.5 mmol/L, and lactate 7.5 mmol/L. Toxicological screening was negative. Cerebrospinal fluid examination revealed an increased cell count with monocyte dominance (126/uL). Computed tomography scan of the head was unremarkable. Fluid attenuated inversion recovery magnetic resonance imaging showed a high intensity signal in the sulci of the left parietal lobe and bilateral occipital lobe (Figures 1(a) and 1(b)). As infectious meningoencephalitis was suspected, empirical therapy was immediately started with meropenem, vancomycin, acyclovir, methyl-prednisolone, and immunoglobulin.

After erythema of the lower leg was noted on day 2, the patient recovered in seven days uneventfully without neurological defect. The erythema was pathologically proved as erythema nodosum. The patient thereafter reported that he had been experiencing recurrent episodes of oral ulcers throughout the last 20 years. Based on the findings including positive human leucocyte antigen (HLA) B-51, clinical manifestations, and the effectiveness of methylprednisolone, the diagnosis of NBD was made and remitted by maintenance of steroid therapy.

## 3. Discussion

Adult NBD happens more in males than in females and commonly between 20 and 40 years of age. Although there are no validated criteria that can be referenced to diagnose NBD, involvement of the central nervous system (CNS) is categorized into parenchymal and nonparenchymal classifications [2]. While the parenchymal type is caused by primary parenchymal neural lesions typically presenting meningoencephalitis, the nonparenchymal type concentrates on the main CNS vascular structures, typically presenting with symptoms and signs attributable to cerebral venous thrombosis [3]. NBD can be acute or chronic based on its differential clinical course.

Some patients with NBD present with both neurological and psychiatric symptoms characterized by paranoid attitudes, euphoria, loss of insight/disinhibition, and bipolar disorders. Depression, anxiety, and somatization are the most frequently reported manifestations [6]. Such a state was recently named Neuro-Psycho-Behçet or Neuropsychiatric Behçet disease [7]. Although the pathogenetic cause behind Neuro-Psycho-Behçet disease has not been resolved, it may be secondary to the involvement of organic neurological factors or associated with poor quality of life and the relapsing/preceding reactivation course of the condition.

Agitation is a frequent presentation in the ED; up to 50% of clinicians are victims of violence during their careers [8]. Patients with prominent psychiatric symptoms may present to psychiatric services during the first instance. However, agitation should be assessed rapidly and managed correctly, since missing a diagnosis of a dangerous etiology may result in severe mortality and morbidity.

A chief goal to treat agitated patient is to protect the patient as well as other patients or medical staff in the area [9]. Verbal deescalation and/or medication are used to avoid dangerous situations in an attempt to reduce restraints [9], which have been shown to be associated with longer hospital stays [10]. In the present case, verbal deescalation strategies were unsuccessful and deep sedative medications were applied to secure safety of the patient and medical staff, otherwise escalating to physical confrontation or resulting in delay in diagnosis and further management. Once the patient is calmed or sedated, obtaining vital signs and a detailed physical examination are important. In patients older than 40, new psychiatric symptoms are more inclined to have an organic than a psychiatric cause [11]. NBD should be considered a possible diagnosis in patients presenting with encephalitis of unknown origin, seizures, psychiatric symptoms, psychosis, or movement disorders. Clinicians should make a diagnosis as fast as possible to ensure well-timed treatment.

At his initial presentation, our patient did not meet International Study Group criteria for the diagnosis of Behçet's disease because he lacked skin and ocular lesions and genital/oral ulcers and had a negative pathergy test [12]. Therefore, we did not initially strongly suspect NBD.

The differential diagnosis of acute agitation with or without fever includes infectious diseases (i.e., sepsis and bacterial, viral, fungal, or tuberculous meningitis), neurologic (i.e., encephalopathy associated with collagen vascular disease or Hashimoto's thyroiditis, autoimmune encephalitis, and paraneoplastic encephalitis), metabolic disorders (i.e., hypo/hyperglycemia, uremia, electrolyte disorders, or thiamine deficiency), substance intoxication/withdrawal, and psychiatric etiologies [1, 13]. In our patient, the combination of positive findings of HLA B-51, repetitive history of oral ulcers, emergence of erythema nodosum, and the effectiveness of steroid therapy raised the definitive diagnosis of NBD.

For patients with an acute psychiatric attack, suspecting an autoimmune mechanism and starting empirical therapy for meningitis may be critical in helping with the diagnosis of NBD. No matter how rapidly management is needed, emergency physicians need to calmly and carefully assess the patient. Importantly, we should be aware that initial recovery from NBD is frequent, but severe impairment also occurs due to relapse. Therefore, careful outpatient follow-up is needed.

## 4. Conclusion

We reported a case of NBD presenting with acute agitation. Altered mental status may be an initial manifestation of acute NBD. A thorough and detailed exam and appropriate treatment strategies with immediate sedation can help emergency clinicians safely and effectively manage these patients.
